# Identification of phlebotomine sand flies (Diptera: Psychodidae) from leishmaniasis endemic areas in southeastern Mexico using DNA barcoding

**DOI:** 10.1002/ece3.5811

**Published:** 2019-11-21

**Authors:** Adebiyi A. Adeniran, Nadia A. Fernández‐Santos, Jorge J. Rodríguez‐Rojas, Nancy Treviño‐Garza, Heron Huerta‐Jiménez, Pedro C. Mis‐Ávila, Wilbert A. Pérez‐Pech, Luis M. Hernández‐Triana, Mario A. Rodríguez‐Pérez

**Affiliations:** ^1^ Laboratorio de Biomedicina Molecular Centro de Biotecnología Genómica Instituto Politécnico Nacional Reynosa México; ^2^ Centro de Investigación y Desarrollo en Ciencias de la Salud Unidad de Patógenos Emergentes, Re-emergentes y Vectores Universidad Autónoma de Nuevo León Nuevo León México; ^3^ Centro Nacional de Programas Preventivos y Control de Enfermedades Secretaria de Salud Mexico City México; ^4^ Laboratorio de Entomología e Insectario Instituto Nacional de Diagnóstico y Referencia Epidemiológicos Secretaria de Salud Mexico City México; ^5^ Servicios de Salud del Estado de Quintana Roo Secretaria de Salud Chetumal México; ^6^ Animal and Plant Health Agency Addlestone UK

**Keywords:** *CO1* gene, DNA barcoding, Mexico, mitochondrial, sand flies

## Abstract

Leishmaniasis, a vector‐borne disease transmitted to humans through the bite of phlebotomine sand flies, is of public health significance in southeastern Mexico. Active and continuous monitoring of vectors is an important aspect of disease control for the prediction of potential outbreaks. Thus, the correct identification of vectors is paramount in this regard. In this study, we employed DNA barcoding as a tool for identifying phlebotomine sand flies collected in localized cutaneous leishmaniasis endemic areas of Quintana Roo, Mexico. Specimens were collected using CDC light and Shannon traps as part of the Mexican Ministry of Health surveillance program. DNA extraction was carried out using a nondestructive protocol, and morphological identification based on taxonomic keys was conducted on slide‐mounted specimens. Molecular taxonomic resolution using the 658‐bp fragment of the mitochondrial cytochrome *c* oxidase subunit 1 (*cox1*) gene was 100% congruent with the morphological identification. Seven species were identified: *Lutzomyia cruciata* (Coquillett 1907), *Lutzomyia longipalpis* (Lutz & Neiva 1912), *Psathyromyia shannoni* (Dyar 1929), *Dampfomyia deleoni* (Fairchild & Hertig 1947), *Dampfomyia beltrani/steatopyga* (Vargas & Díaz‐Nájera 1951), *Bichromomyia olmeca olmeca* (Vargas & Díaz‐Nájera, 1959), and *Brumptomyia mesai* (Sherlock 1962). Mean intraspecific divergence ranged from 0.12% to 1.22%, while interspecific distances ranged from 11.59% to 19.29%. Neighbor‐joining (NJ) analysis using the Kimura 2‐parameter model also showed specimens of the same species to be clustered together. The study provides the first *cox1* sequences for three species of sand flies and indicates the utility of DNA barcoding for phlebotomine sand flies species identification in southeastern Mexico.

## Graphical abstract

Correct identification of vectors is paramount to control, and we employed the DNA barcoding as a tool for identifying phlebotomine sand flies collected in leishmaniasis endemic areas of Quintana Roo, Mexico. We provide the first *cox1* sequences for three species of sand flies and indicate the utility of DNA barcoding for phlebotomine sand flies species identification in Mexico.

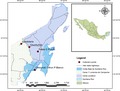

## 1 INTRODUCTION

Over half of tropical infectious diseases are vector‐borne, with arthropods directly or indirectly involved in the transmission of pathogens to humans (WHO, 2017). Leishmaniasis, vectored by phlebotomine sand flies, is of significant public health importance in southern Mexico. It is an endemic disease in this part of the country with localized cutaneous leishmaniasis as the predominant clinical form of the disease (Ready, 2013; Velasco‐Castrejón, Ibáñez‐Bernal, & Rivas‐Sánchez, 1994). Other forms such as diffuse cutaneous leishmaniasis, mucocutaneous leishmaniasis, and visceral leishmaniasis are, however, not uncommon (Velasco‐Castrejón et al., 1994). Leishmaniasis control is often exacerbated by the complexity of the transmission cycle that involves several vectors and reservoir hosts, depending on geographical locations (Monroy‐Ostria, Hernandez‐Montes, & Barker, 2000).

There are ca. 1,000 described species of phlebotomine sand flies in the world with 530 species known in the Americas (Shimabukuro, de Andrade, & Galati, 2017). Of these, 51 species are present in Mexico with 26 species in the Yucatan Peninsula, and eleven of these of suggested medical importance (Cohnstaedt, Beati, Caceres, Ferro, & Munstermann, 2011; González et al., 2011; Ibáñez‐Bernal, 2000). *Lutzomyia* (*Lutzomyia*) *longipalpis* and *Pintomyia* (*Pifanomyia*) *evansi* (Nuñez‐Tovar 1924) are the principal vectors of visceral leishmaniasis caused by *Leishmania infantum* (Nicolle 1908; Ibáñez‐Bernal, Rodríguez‐Domínguez, Gómez‐Hernández, & Ricardez‐Esquinca, 2004; Mauricio, Howard, Stothard, & Miles, 1999). Although *Bichromomyia olmeca olmeca* has been argued as the only competent vector of *Leishmania mexicana* (Biagi 1953), the causative agent of cutaneous leishmaniasis (González et al., 2011), *Lutzomyia* (*Tricholateralis*) *cruciata*, *Psathyromyia* (*Psathyromyia*) *shannoni*, *Nyssomyia ylephiletor* (Fairchild & Hertig 1952), and *Psychodopygus panamensis* (Shannon 1926) have also been hypothesized to be vectors in the Yucatan Peninsula (Pech‐May, Escobedo‐Ortegon, Berzunza‐Cruz, & Rebollar‐Téllez, 2010; Pech‐May et al., 2016; Sánchez‐García, Berzunza‐Cruz, Becker‐Fauser, & Rebollar‐Téllez, 2010). *Lutzomyia* (*Tricholateralis*) *gomezi* (Nitzulescu 1931), *Lutzomyia* (*Tricholateralis*) *diabolica* (Hall 1936), *Pintomyia* (*Pifanomyia*) *ovallesi* (Ortiz 1952), and *Dampfomyia* (*Dampfomyia*) *anthophora* (Addis 1945) are also suspected vectors of cutaneous leishmaniasis (Bonfante‐Garrido, Spinetti, Cupillo, Momen, & Grimaldi, 1991; Endris, Young, & Perkins, 1987).

Unambiguous species identification is necessary to ascertain the role of each species in disease transmission (Cohnstaedt et al., 2011). However, uncertainties about the taxonomic resolution of certain groups are common as morphological identification is based on gender‐specific morphological traits. This makes identification often difficult because of isomorphism among phlebotomine sand fly of different species of the same sex, and because of the presence of species complexes (Cohnstaedt et al., 2011; Hebert, Cywinska, Ball, & de Waard, 2003; Testa, Montoya‐Lerma, Cadena, Oviedo, & Ready, 2002). Furthermore, ecological niche modeling has suggested incongruency in vectors and disease distributions in Mexico (González et al., 2010), necessitating a review of vector identification techniques. Molecular identification techniques using standard mitochondrial markers have become a popular approach, especially the use of cytochrome *c* oxidase subunit I (*cox1*) for DNA barcoding (Hebert, Cywinska, et al., 2003). In this study, we present evidence of the suitability of the DNA barcoding approach to support phlebotomine sand fly identification in Mexico. We used the DNA barcode variability as a tool for molecular taxonomy in local sand fly population in southeast Mexico and provide baseline data towards the establishment of a phlebotomine sand fly barcode reference library in Mexico. The genetic relationship with other phlebotomine sand fly sequences from the new world was also investigated.

## 2 MATERIALS AND METHODS

### 2.1 Study area

**Figure 1:**
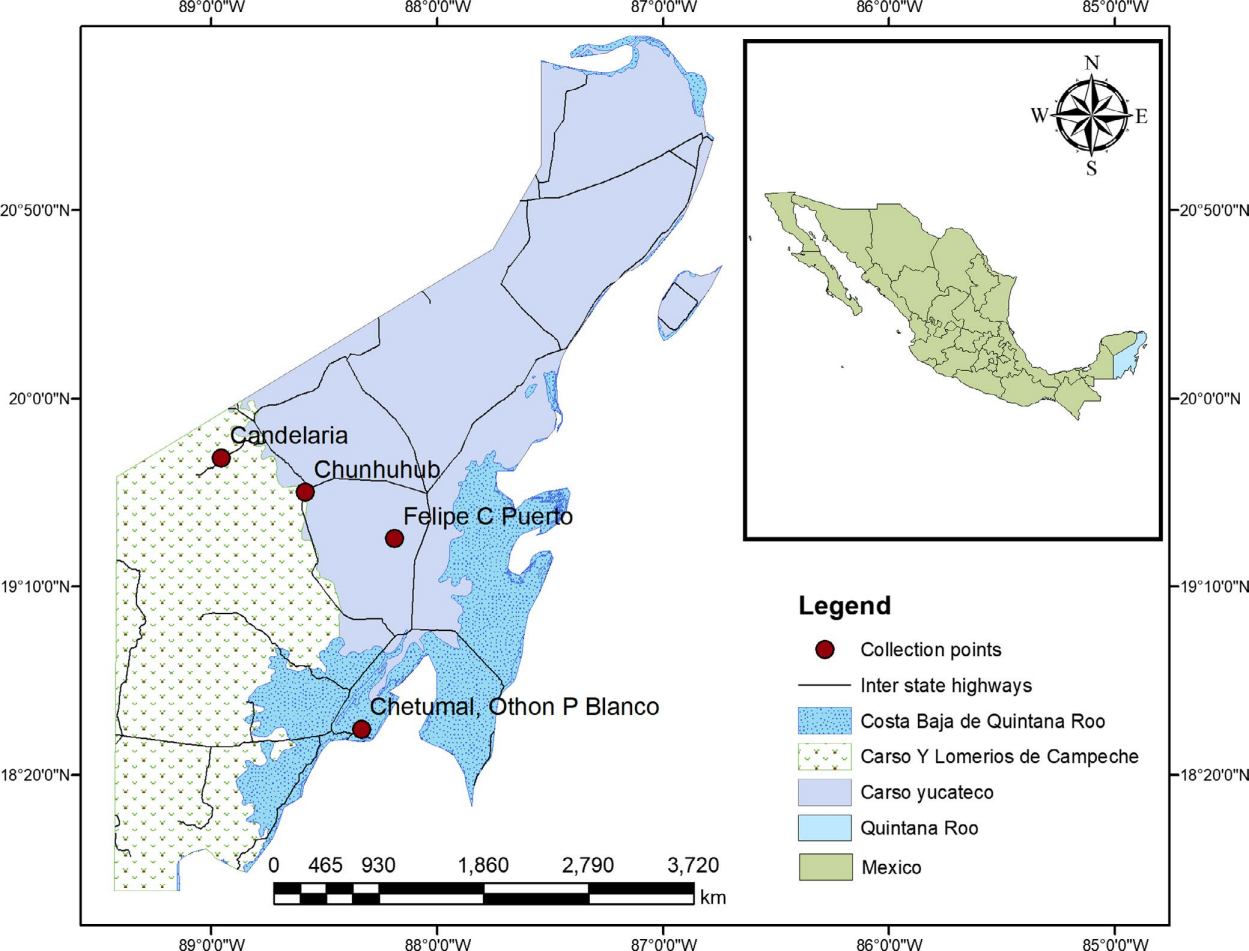
Map of study area showing sampling locations

Quintana Roo is one of the 31 federating units of Mexico. It is located on the southeastern most part of the country sharing boundaries with Yucatan, Gulf of Mexico, Caribbean Sea, and Belize. It lies between the latitude 19°36′N and longitude 87°55′W. The state has three physiographic provinces that include the *Costa Baja de Quintana Roo*, *Carso y Lomerios de Campeche* and *Carso Yucateco* which are representative of the Yucatan Peninsula (Barrera‐Marin, 1964). Samples were collected in four localities from leishmaniasis endemic areas in Quintana Roo: (a) Chetumal Othon P. Blanco (18°32′4.2″N, 88°20′7.08″W), (b) Candelaria or Jose Maria Morelos (19°44′11.04″N, 88°57′23.4″W), (c) Chunhuhub (19°35′7.8″N, −88°35′3.48″W), and (d) Felipe Carrillo Puerto (19°22′42.96″N, 88°11′21.12″W; Figure 1). These areas are regularly monitored for the presence of leishmaniasis by the Ministry of Health in coordination with the Centro Nacional de Programas Preventivos y Control de Enfermedades (CENAPRECE).

### 2.2 Sample collection and identification

Samples were collected by the state health authorities as part of an entomological surveillance for monitoring transmission of diseases between October 2016 and February 2018 using CDC light and Shannon traps. Samples were stored in 70% ethanol and at −20°C prior to molecular processing. Species identification was carried out at the Centro de Investigación y Desarrollo en Ciencias de la Salud, Universidad Autónoma de Nuevo León (CIDICS‐UANL) after DNA extraction. Phlebotomine sand flies were clarified and mounted in Euparal^®^ (Bioquip Products, Inc.) as permanent slide as described by Young and Duncan (1994) and Ibañez‐Bernal (2005a). Morphological identification was carried out using published dichotomous keys (Ibañez‐Bernal, 2005a, 2005b; Young & Duncan, 1994), phylogenetic classification of Galati (1995, 2016), and the abbreviations for genera and subgenera proposed by Marcondes, (2007). Voucher specimens were deposited in the arthropod collection of CIDICS‐UANL.

### 2.3 DNA extraction, PCR, and sequencing

Genomic DNA extraction was carried out using a slightly modified nondestructive DNA extraction method as described by Truett et al. (2000). Briefly, whole insect bodies were put directly into individual 200 μl PCR tubes containing 20 μl of alkaline lysis buffer and frozen at −20°C for 5–6 hr. Afterward, the tubes were incubated in a PCR thermocycler for 30 min at 94°C and 4°C for 5 min to cool down. Samples were vortexed gently using the Genie 2 Vortex Mixer (Daigger Scientific), and 20 μl of the neutralizing buffer was added. Samples were then spun briefly and stored at −80°C overnight for another freeze–thaw cycle before PCR processing. Insect samples were removed, put back in ethanol, and stored for morphological identification.

The 658‐bp fragment of the *cox1* gene was amplified using the primers LCO1490 and HCO2198 (Folmer, Black, Hoeh, Lutz, & Vrijenhoek, 1994) and a previously described PCR protocol (Hernández‐Triana et al., 2012). PCR reactions were performed in a total volume of 20 μl using 2 μl of DNA extract, 1 × NH_4_ buffer, 2 pmol/μl dNTPs, 1.5 mM MgCl_2_, 10 pmol/μl of each primer, 0.6 U *Taq* DNA polymerase (Bioline), and 20 mg/ml bovine serum albumin. The reaction cycle consisted of an initial 1 min at 94°C, followed by a preamplification 5 cycles of 94°C for 1 min, 45°C for 1.5 min, 72°C for 1.5 min, an amplification step of 35 cycles of 94°C for 1 min, 57°C for 1.5 min, 72°C for 1.5 min with a final extension of 72°C for 5 min. PCR products were separated by electrophoresis in 1.5% agarose gel, and samples showing correct band size were purified using the QIAquick PCR purification kit and sequenced in both directions using the ABI PRISM^®^ BigDye^®^ Terminator sequencing kit (Applied Biosystems) at commercial sequencing facilities with the same primer pair.

### 2.4 Sequence analysis

DNA sequences generated in both directions were edited manually using BioEdit sequence alignment Editor v.7.0.5.3 (Hall, 1999) and consensus sequences generated using the in‐built ClustalW (Larkin et al., 2007). Multiple sequence alignment, base pair content, and coding positions analysis were completed in MEGA v.7 (Kumar, Stecher, & Tamura, 2016). Mean genetic distances, pairwise sequence divergences, and neighbor‐joining (NJ) analysis were calculated using the Kimura 2‐parameter (K2P) distance model with 1,000 bootstrap replicates (Saitou & Nei, 1987). The choice of K2P was to make results comparable with other DNA barcoding studies and because it provides conservative estimates of long branches than other models as it underestimates the number of multiple hits (Nei & Kumar, 2000). The number of haplotypes, polymorphic sites, and nucleotide diversity were determined using DnaSP v6 (Rozas et al., 2017).

A dataset of 156 sequences containing the 44 *cox1* sequences generated from this study, 3 outgroups (*Aedes aegypti* Linnaeus 1762, *Culex quinquefasciatus* Say 1823, and *Gigantodax antarcticus* Bigot 1888), and 109 sand flies *cox1* sequences, including species reported in Mexico, downloaded from BOLD (http://www.boldsystems.org) and GenBank, was created and used in the analyses (Ibáñez‐Bernal, 1999, 2000, 2001, 2003). Sequences that were submitted to databases from similar barcoding studies were given higher consideration over those not from a DNA barcoding study, and sequences less than 500 bp were excluded (Table S1).

Molecular species delimitation was accomplished using the Automatic Barcode Gap Discovery (ABGD) software (Puillandre, Lambert, Brouillet, & Achaz, 2012). The minimum intraspecific distance (*P*_min_) and maximum intraspecific distance (*P*_max_) were limited to the default of 0.001 and 0.1, respectively, with the default barcode gap width of 1.5 and K2P model.

## 3 RESULTS

A total of 50 phlebotomine sand flies specimens (35 males and 15 females) representing collections from 4 localities were used in the study, although we only succeeded in obtaining sequences from 44 samples (33 males and 11 females). Five genera (*Dampfomyia*, *Bichromomyia*, *Brumptomyia*, *Lutzomyia*, and *Psathyromyia*) and seven species including *Lu.* (*Trl.*) *cruciata*, *Lu.* (*Lut.*) *longipalpis*, *Pa.* (*Psa.*) *shannoni*, *Dampfomyia* (*Coromyia*) *deleoni*, *Dampfomyia* (*Cor.*) *beltrani/steatopyga*, *Bi. olmeca olmeca*, and *Brumptomyia mesai* were identified (Table 1). Species discrimination of *Da. beltrani*/*Da. steatopyga* could not be accomplished because male specimens, which are needed to separate these species, were not collected in the present study.

**Table 1:** List and location of phlebotomine sand flies analyzed in this study

Species	Gender	Collection site	Date of collection	BLAST result (% identity)^a^	GenBank accession ID
1. *Bichromomyia olmeca olmeca*	F	Chetumal Othon P. Blanco	Feb 2018	*Nyssomyia yuilli yuilli* (Young & Porter, 1972) (90.0)	MK851274
2. *Brumptomyia mesai*	F	Chetumal Othon P. Blanco	Feb 2018	*Brumptomyia hamata* (Fairchild & Hertig, 1947) (96.5)	MK851243
3. *Brumptomyia mesai*	F	Chetumal Othon P. Blanco	Feb 2018	*Br. hamata* (97.4)	MK851242
4. *Brumptomyia mesai*	F	Chetumal Othon P. Blanco	Feb 2018	*Br. hamata* (97.7)	MK851244
5. *Dampfomyia beltrani/ steatopyga*	F	Chetumal Othon P. Blanco	Feb 2018	*Phlebotomus longicuspis* (Nitulescu, 1930) (85.7)	MK851245
6. *Dampfomyia beltrani*/*steatopyga*	F	Chetumal Othon P. Blanco	Feb 2018	*Micropygomyia venezuelensis* (Floch & Abonnenc, 1948) (85.6)	MK851246
7. *Dampfomyia deleoni*	M	Candelaria	Oct 2016	*Lutzomyia renei* (Martins, Falcão & Silva, 1957) (89.1)	MK851251
8. *Dampfomyia deleoni*	M	Candelaria	Oct 2016	*Lu. renei* (89.0)	MK851252
9. *Dampfomyia deleoni*	M	Candelaria	Oct 2016	*Lu. renei* (88.6)	MK851253
10. *Dampfomyia deleoni*	M	Candelaria	Oct 2016	*Lu. renei* (89.1)	MK851249
11. *Dampfomyia deleoni*	M	Candelaria	Oct 2016	*Lu. renei* (89.1)	MK851250
12. *Lutzomyia cruciata*	F	Chetumal Othon P. Blanco	Feb 2018	*Lu. cruciata* (98.9)	MK851248
13. *Lutzomyia cruciata*	M	Candelaria	Oct 2016	*Lu. cruciata* (98.1)	MK851247
14. *Lutzomyia longipalpis*	M	Chunhuhub	Oct 2016	*Lu. longipalpis* (93.4)	MK851267
15. *Lutzomyia longipalpis*	M	Chunhuhub	Oct 2016	*Lu. longipalpis* (93.7)	MK851266
16. *Lutzomyia longipalpis*	M	Chunhuhub	Oct 2016	*Lu. longipalpis* (93.4)	MK851265
17. *Lutzomyia longipalpis*	M	Chunhuhub	Oct 2016	*Lu. longipalpis* (93.6)	MK851254
18. *Lutzomyia longipalpis*	M	Chunhuhub	Oct 2016	*Lu. longipalpis* (93.4)	MK851264
19. *Lutzomyia longipalpis*	M	Chunhuhub	Oct 2016	*Lu. longipalpis* (93.7)	MK851263
20. *Lutzomyia longipalpis*	M	Chunhuhub	Oct 2016	*Lu. longipalpis* (93.9)	MK851262
21. *Lutzomyia longipalpis*	M	Chunhuhub	Oct 2016	*Lu. longipalpis* (93.2)	MK851261
22. *Lutzomyia longipalpis*	M	Chunhuhub	Oct 2016	*Lu. longipalpis* (93.9)	MK851260
23. *Lutzomyia longipalpis*	M	Chunhuhub	Oct 2016	*Lu. longipalpis* (93.4)	MK851259
24. *Lutzomyia longipalpis*	M	Chunhuhub	Oct 2016	*Lu. longipalpis* (93.1)	MK851258
25. *Lutzomyia longipalpis*	M	Candelaria	Oct 2016	*Lu. longipalpis* (93.7)	MK851257
26. *Lutzomyia longipalpis*	M	Felipe C Puerto	Oct 2016	*Lu. longipalpis* (93.6)	MK851256
27. *Lutzomyia longipalpis*	M	Felipe C Puerto	Oct 2016	*Lu. longipalpis* (93.7)	MK851255
28. *Lutzomyia longipalpis*	M	Felipe C Puerto	Oct 2016	*Lu. longipalpis* (93.4)	MK851273
29. *Lutzomyia longipalpis*	M	Felipe C Puerto	Oct 2016	*Lu. longipalpis* (93.4)	MK851272
30. *Lutzomyia longipalpis*	M	Felipe C Puerto	Oct 2016	*Lu. longipalpis* (93.4)	MK851271
31. *Lutzomyia longipalpis*	M	Felipe C Puerto	Oct 2016	*Lu. longipalpis* (93.4)	MK851270
32. *Lutzomyia longipalpis*	M	Felipe C Puerto	Oct 2016	*Lu. longipalpis* (93.7)	MK851269
33. *Lutzomyia longipalpis*	M	Felipe C Puerto	Oct 2016	*Lu. longipalpis* (93.6)	MK851268
34. *Psathyromyia shannoni*	M	Chetumal Othon P. Blanco	Feb 2018	*Pa. shannoni* (99.3)	MK851284
35. *Psathyromyia shannoni*	M	Chetumal Othon P. Blanco	Feb 2018	*Pa. shannoni* (99.5)	MK851275
36. *Psathyromyia shannoni*	M	Chetumal Othon P. Blanco	Feb 2018	*Pa. shannoni* (99.7)	MK851276
37. *Psathyromyia shannoni*	F	Chetumal Othon P. Blanco	Feb 2018	*Pa. shannoni* (100.0)	MK851277
38. *Psathyromyia shannoni*	M	Chetumal Othon P. Blanco	Feb 2018	*Pa. shannoni* (99.7)	MK851278
39. *Psathyromyia shannoni*	F	Chetumal Othon P. Blanco	Feb 2018	*Pa. shannoni* (100.0)	MK851280
40. *Psathyromyia shannoni*	F	Chetumal Othon P. Blanco	Feb 2018	*Pa. shannoni* (99.4)	MK851281
41. *Psathyromyia shannoni*	F	Chetumal Othon P. Blanco	Feb 2018	*Pa. shannoni* (98.9)	MK851283
42. *Psathyromyia shannoni*	M	Chetumal Othon P. Blanco	Feb 2018	*Pa. shannoni* (99.5)	MK851282
43. *Psathyromyia shannoni*	M	Chetumal Othon P. Blanco	Feb 2018	*Pa. shannoni* (99.4)	MK851279
44. *Psathyromyia shannoni*	M	Chetumal Othon P. Blanco	Feb 2018	*Pa. shannoni* (99.5)	MK851285

Abbreviation: F, female; M, male.

a BLAST result is as it is at the time of query (August 2019).

The 44 *cox1* sequences generated were uploaded to the BOLD database (http://www.boldsystems.org) under the project “AAASF,” and the sequences were also submitted to GenBank (accession numbers MK851242–MK851285). Final alignment of the 44 sequences obtained was 654 bp with 354 variable nucleotide positions, 234 conserved sites, and 91 parsimony informative sites. There was no stop codon, insertions, or deletions observed suggesting the absence of nuclear pseudogenes of mitochondrial origin (NUMTs). The average nucleotide compositions of the *cox1* sequences were 37.5% T, 28.4% A, 18% C, and 16.1% G with mean AT richness of 65.9%. Individual species were represented between one and twenty individuals. All sequences had more T in the second and third codons than the first (Table S2). The overall mean genetic distance was 11.06%, and pairwise Kimura 2‐parameter genetic distance ranged from 0% to 19.8% (Table S3). Intraspecific mean sequence divergence ranged between 0.12% and 1.22% (Appendix S1), while interspecific divergence ranged from 11.59% to 19.29% (Appendix S2). When the sequences obtained in this study were analyzed together, the highest intraspecific mean genetic distance of 1.22% was found in *Lu. cruciata*, followed by *Pa. shannoni* (1.13%). Also, 23 haplotypes were generated with a range of 1–8 haplotypes per species (Appendix S1). However, higher sequence divergence was observed when our dataset was compared with the other sand flies sequences from the new world downloaded from BOLD and GenBank. Mean intraspecific divergence ranged from 0% to 9.48%, with the highest divergence (9.48%) also found in *Lu. cruciata* (Table 2). High intraspecific divergence was also found in *Br. mesai* (9.12%), *Pa. shannoni* (5.47%), and *Lu. longipalpis s.l* (4.51%). Interspecific divergence ranged from 6% to 22.2% with the highest divergence between *Psathyromyia* (*Forattiniella*) *carpenteri* (Fairchild & Hertig 1953) and *Da. beltrani*/*Da. steatopyga* (Table 3).

**Table 2:** List of sand flies species, country of collection, and number of specimens with DNA barcodes

Species	Country	Number of sequences (*n*)	Mean sequence divergence (%)	Maximum pairwise divergence (%)
1. *Bichromomyia flaviscutellata* (Mangabeira, 1942)	Brazil	2	0.30	0.30
2. *Bichromomyia olmeca bicolor* (Fairchild & Theodor 1971)	Colombia	1	—	—
3. *Bichromomyia olmeca olmeca*	Mexico	1	—	—
4. *Brumptomyia hamata*	Colombia	2	0.00	0.00
5. *Brumptomyia mesai* b	Mexico, Colombia	5	9.12^a^	15.56
6. *Dampfomyia beltrani*/*steatopyga*	Mexico	2	0.61	0.61
7. *Dampfomyia deleoni*	Mexico	5	0.34	0.61
8. *Lutzomyia cruciata* b	Mexico, Honduras	3	9.48	13.62
9. *Lutzomyia gomezi*	Honduras	3	0.32	0.48
10. *Lutzomyia* (*Helcocyrtomyia*) *hartmanni* (Fairchild & Hertig 1957)	Colombia	4	0.38	0.61
11. *Lutzomyia longipalpis s.l.* a ^,^ b	Mexico, Brazil, Colombia, Honduras	40	4.51	9.24
12. *Micropygomyia* (*Micropygomyia*) *cayennensis cayennensis* (Floch & Abonnenc 1941)	Colombia	5	0.95	1.54
13. *Micropygomyia* (*Sauromyia*) *trinidadensis* (Newstead 1922)	Brazil	4	1.43	2.06
14. *Pintomyia evansi*	Honduras	1	—	—
15. *Pintomyia ovallesi*	Costa Rica	2	0.35	0.35
16. *Pintomyia* (*Pifanomyia*) *serrana* (Damasceno & Arouck 1949)	Colombia, Peru	4	0.13	0.46
17. *Psathyromyia abonnenci*	Colombia	3	0.61	0.92
18. *Psathyromyia carpenteri*	Colombia	1	—	—
19. *Psathyromyia shannoni* a	Brazil, Mexico, USA, Colombia	56	5.47	12.20
20. *Psychodopygus panamensis*	Ecuador	5	1.74	2.90
21. *Trichopygomyia triramula* (Fairchild & Hertig 1952)	Colombia, Ecuador	4	1.50	2.03

Note

Mean (%) intraspecific values of sequence divergence (Kimura 2‐parameter distance) are shown with missing entries indicating that <2 specimens were analyzed.

a Species complexes.

b Taxa with above 2% distance divergence.

**Table 3:** Percentage interspecific pairwise K2P genetic divergence of DNA barcodes of sand flies species analyzed in the current study

	1	2	3	4	5	6	7	8	9	10	11	12	13	14	15	16	17	18	19	20	21
1. *Pa. abonnenci*																					
2. *Pa. shannoni*	8.6																				
3. *Ps. panamensis*	15.2	13.7																			
4. *Mi. trinidadensis*	16.9	15.9	14.2																		
5. *Ty. triramula*	18.0	16.8	14.8	15.2																	
6. *Pi. serrana*	17.8	17.4	13.6	15.0	15.6																
7. *Pi. ovallesi*	18.7	17.5	19.6	16.7	15.9	18.0															
8. *Bi. olmeca olmeca*	17.2	15.4	13.7	16.9	15.6	16.1	16.9														
9. *Lu. longipalpis*	16.8	15.3	13.1	16.0	15.3	14.6	16.6	14.6													
10. *Lu. hartmanni*	19.7	18.8	16.2	15.7	16.6	15.4	13.9	16.5	15.0												
11. *Lu. gomezi*	17.6	16.8	16.3	18.7	18.5	17.7	17.7	16.6	17.9	17.4											
12. *Pi. evansi*	19.0	18.0	18.0	14.6	13.8	15.2	11.8	15.2	14.8	13.5	17.0										
13. *Da. deleoni*	19.2	17.1	14.5	15.8	14.1	14.7	19.6	14.6	15.5	16.9	17.8	14.9									
14. *Lu. cruciata*	17.8	16.6	16.0	17.7	16.3	15.8	17.0	16.2	14.1	15.4	15.8	16.7	17.2								
15. *Mi. cayennensis cayennensis*	18.9	18.1	15.6	15.5	17.2	17.6	17.1	17.8	15.9	18.1	18.2	17.4	17.2	15.9							
16. *Pa. carpenteri*	15.7	14.9	12.8	17.4	16.6	15.7	17.4	14.9	14.2	14.9	18.7	16.7	17.5	17.4	14.9						
17. *Da. beltrani/ Da. steatopyga*	22.2	20.0	19.9	18.2	19.1	18.1	20.5	18.1	20.4	20.5	19.7	18.8	15.3	19.5	20.4	20.2					
18. *Br. mesai*	19.3	18.0	15.3	16.5	16.4	15.5	17.2	17.5	15.1	16.4	18.2	15.4	16.8	16.6	17.3	15.2	19.1				
19. *Br. hamata*	19.2	18.1	14.5	17.3	16.6	16.9	17.2	16.6	14.8	17.0	19.0	15.4	16.6	17.1	17.1	14.7	17.6	6.9			
20. *Bi. flaviscutellata*	16.1	14.8	12.9	14.5	18.0	13.3	17.7	13.6	13.2	17.7	17.8	17.1	16.3	16.7	16.1	14.0	19.1	17.2	17.3		
21. *Bi. olmeca bicolor*	18.5	17.1	14.9	14.4	17.3	15.1	17.6	14.9	16.9	15.8	19.5	16.1	18.0	18.2	16.1	14.6	20.4	18.8	20.3	14.2	

**Figure 2:**
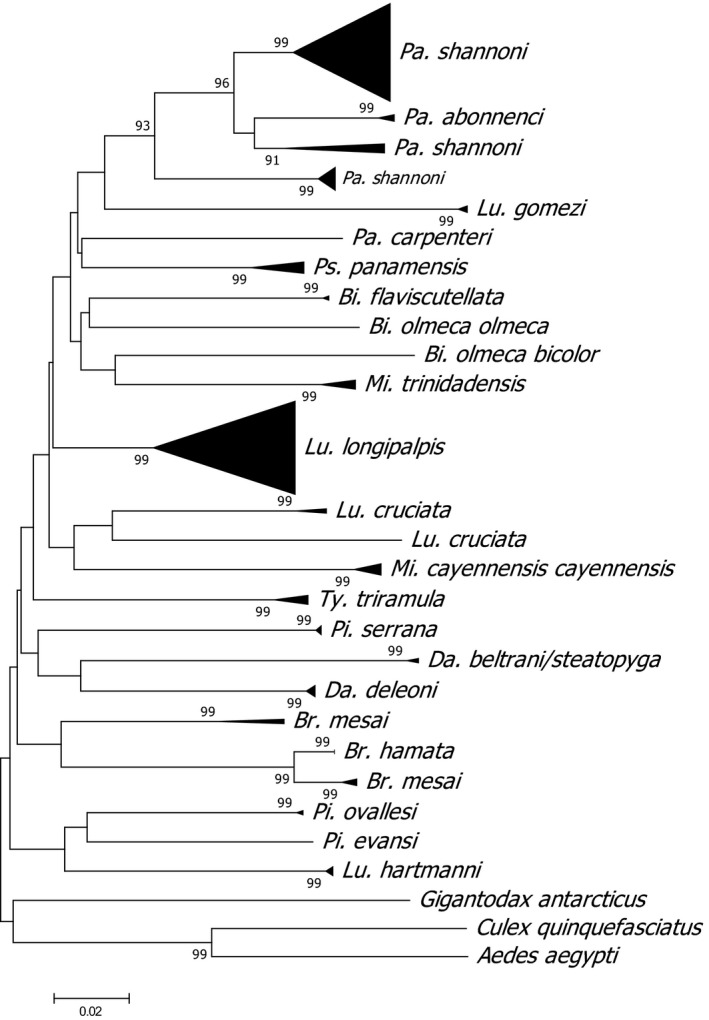
Bootstrapped neighbor‐joining (NJ) tree with 1,000 replicas showing the clustering pattern of sand flies species based on the barcoding region of the mitochondrial *cox1* gene. Expanded tree is shown in Figure S1

The NJ tree using the 156 *cox1* sequences dataset shows that conspecific individuals clustered together in most cases with high bootstrap support, and there was a clear separation among congeneric species (Figure 2). However, *Psathyromyia* (*Psathyromyia*) *abonnenci* (Floch & Chassignet 1947) specimens clustered with *Pa. shannoni* and two separate clades of *Br. mesai* were observed. Furthermore, *Lu. longipalpis s.l.*, *Lu. cruciata*, and *Pa. shannoni* showed a deep split in the NJ tree which agrees with the high intraspecific genetic divergence observed in these taxa (Table 2).

Using the default ABGD settings, nine potential barcode gaps were identified with two without recursive partitions from the 44 sequences generated in the present study (Appendix S3). Barcode gap with prior intraspecific divergence values between 1% and 2.5% was considered for this study, to enable comparison with other barcoding studies and allow the use of the lower limit of the 2%–3% (Hebert, Cywinska, et al., 2003). Two values of barcode gaps were found within this range: 1.29% and 2.15%, and even though the initial partition in both values grouped species into 7, the recursive partition under both values partitioned the species into eight groups. All species were recognized by the ABGD partitioning as they all grouped homogenously except *Pa. shannoni* that split into two groups. Similarly, eight BINs (BOLD:ADP3520, BOLD:ADQ1943, BOLD:ADW1198, BOLD:AAY4824, BOLD:AAY4825, BOLD:AAY5017, BOLD:ACT 9235, and BOLD:ADU0036) were assigned by BOLD for the sequences representing seven species encountered in this study, with three of these (BOLD:ADP3520 for *Lu. cruciata*, BOLD:ADW1198 for *Bi. olmeca*, and BOLD:ADQ1943 for *Da. beltrani/Da.steatopyga*) new to BOLD. There were no shared BINs among species; however, *Pa. shannoni* is represented by two BINs, BOLD:AAY4824 and BOLD:AAY4825.

## 4 DISCUSSION

The fundamental aim of DNA barcoding is to standardize molecular approach used in complementing morphological species identification, and this has been previously exploited in phlebotomine sand flies (Arrivillaga, Norris, Feliciangeli, & Lanzaro, 2002; Azpurua, De La Cruz, Valderama, & Windsor, 2010; Gutiérrez, Vivero, Vélez, Porter, & Uribe, 2014). Here, we present preliminary information on the utility of the DNA barcoding approach to support the identification of phlebotomine sand fly in leishmaniasis endemic communities in Mexico. Forty‐four specimens collected during routine epidemiological phlebotomine sand flies surveillance revealed seven species including *Lu. cruciata*, *Lu. longipalpis*, *Pa. shannoni*, *Da. deleoni*, *Da. beltrani*/*Da. steatopyga*, *Bi. olmeca olmeca*, and *Br. mesai*. Eighteen (~35%) of 51 phlebotomine sand fly species registered in Mexico (Ibáñez‐Bernal, 2000, 2002, 2003; Rosete‐Ortiz et al., 2011) have a *cox1* barcode sequence represented in BOLD database (Table 2). Prior to this study, no previous attempt has been made to investigate the utility of DNA barcoding to identify sand flies in Mexico. Florin and Rebollar‐Téllez (2013) utilized the *cox1* marker to investigate the genetic divergence between *Pa. shannoni* populations in Mexico and USA, but barcoding was not with the main objective of the study.

Phlebotomine sand flies have been shown to exhibit A‐T bias in their nucleotide composition, and the 66% A‐T composition in this study is consistent with similar results in Latin America (Azpurua et al., 2010; Contreras Gutiérrez, Vivero, Vélez, Porter, & Uribe, 2014; de Pinto et al., 2015) and India (Kumar, Srinivasan, & Jambulingam, 2012). We obtained a coherent matrix of DNA barcode sequences that differentiated all species collected without ambiguous identification. High interspecific divergence (>3%) was observed in both datasets, and these agree with the interspecific limit for insects as proposed by Hebert, Ratnasingham, & de Waard, 2003). Sequences from the seven species from the current study had a mean intraspecific divergence of <2% (Appendix S1) that is also within proposed limit of species for barcode studies (Hebert, Cywinska, et al., 2003). However, although a low mean intraspecific divergence was observed among sequences generated from the present study (Appendix S2), a much higher mean intraspecific divergence was observed in *Lu. longipalpis*, *Lu. cruciata*, *Pa. shannoni*, and *Br. mesai* when compared with sequences from other countries (Table 2). This could be due to varying geographical locations suggesting population differentiation, presence of cryptic species (Gutiérrez et al., 2014), and/or possible cases of misidentification of the specimens of the *cox1* sequences retrieved from GenBank, the latter being a more plausible explanation given that some of these *cox1* sequences retrieved from GenBank were from unpublished studies (Table S1).

The intraspecific variability of the *Lu. longipalpis s.l.* population in the present study, though suggesting homogeneity with a mean divergence of 0.39%, and a maximum pairwise divergence of ~1%, produced eight haplotypes (Appendix S1) from two localities (Chunhuhub and Felipe C. Puerto). However, a higher divergence (4.51%) was observed when analyzed with sequences from Brazil, Honduras, and Colombia forming three clades in the NJ analysis (Figure S1). This is consistent with extant literature that *Lu. Longipalpis s.l.* is a species complex that exhibits a complex population structure (Maingon, Ward, Hamilton, Bauzer, & Peixoto, 2008; de Pinto et al., 2015; Souza, Brazil, & Araki, 2017). This is particularly shown in a complex grouping pattern of *Lu. longipalpis* with *Lutzomyia. cruzi* (Mangabeira, 1938) in a study in Brazil (de Pinto et al., 2015), which supports the hypothesis of recent speciation events in the taxon (Souza et al., 2017).

High intraspecific divergence was also observed in *Lu. cruciata* and *Pa. shannoni*, which are also of potential medical importance in the Yucatan Peninsula (Pech‐May et al., 2010, 2016). *Pa. shannoni* is a well‐established species in Mexico with recent report of population divergence in southern Mexico (Florin & Rebollar‐Téllez, 2013). Our material originates from the same locality (Chetumal Othon P. Blanco), and our results supported this hypothesis with a high maximum pairwise divergence of 4.5% (Appendix S1) and a deep split in the NJ tree (Figure 2). This taxon is also the only one that groups with two partitions in ABGD analysis and has two BINs (BOLD:AAY4824 and BOLD:AAY4825) assigned. We, however, observed an intraspecific mean divergence of 1.13% that is within the established limit for species delimitation in barcoding studies (Hebert, Cywinska, et al., 2003; de Pinto et al., 2015) with relatively higher number of samples (*n* = 11). Availability of male and female samples in our materials also eliminated doubts of misidentification (Cohnstaedt et al., 2011; Florin & Rebollar‐Téllez, 2013). Furthermore, there were no ambiguities in the NCBI BLAST analysis of sequences generated in this study (Table 1). This study confirms the presence of cryptic diversity involving two populations of *Pa. shannoni* in Othon P Blanco, Quintana Roo. Morphological revision of the Shannoni group of the genus *Psathyromyia* (Barretto, 1962) resurrected *Psathyromyia bigeniculata* (Floch & Abonnenc 1941) and *Psathyromyia limai* (Fonseca 1935) from the synonymy of *Pa. shannoni*, and *Psathyromyia pestanai* (Barretto & Coutinho 1941) was proposed as a new junior synonym of *Pa. limai* (Sábio, Andrade, & Galati, 2014). Although *Pa. bigeniculata* and *Pa. limai* were identified by de Pinto et al. (2015) in their study, these species were identified as *Pa. shannoni* on GenBank database, complicating the taxonomic identity of members within this species complex. As our sequence grouped separately from *Pa. abonnenci*, another closely related species of *Pa. shannoni* (Figure 2), the true identity of members of the species complex encountered in this study is unclear. Thus, detailed morphological and molecular investigation of this species group in Quintana Roo and southern Mexico, using other genetic markers and larger sample population, might be required to ascertain the composition of this complex. In addition, given that the vectorial competence of this species is still unresolved and the potential effect on the epidemiology of leishmaniasis in this endemic area is unknown (Bennett et al., 2002), this is an important issue for future research.

Although NJ analysis is essentially not a phylogenetic tool, it is an appropriate method for evaluating distances when combined with bootstrap analysis (Felsenstein, 1985). All individuals belonging to the same species grouped together and were supported by high bootstrap values. Congeneric groupings were also well‐separated in the NJ tree supporting our morphological identifications. Although cases of misidentification in DNA barcoding studies are not uncommon, this could have serious implications for end users of reference libraries (Collins & Cruickshank, 2013; Hernández‐Triana et al., 2019). It appears that the incongruence observed in the NJ analysis for *Pa. abonnenci* (Figure 2) seems to be one of such a case. However, the inability to reidentify the vouchers specimens from which the sequences were generated due to lack of access and unavailability of *Pa. abonnenci* sequences from the current study does not allow us to make further comments on its identity. We believe, however, that these are separate species based on the clear interspecific divergence of 8.6% found between the *Pa. shannoni* and *Pa. abonnenci* sequences we analyzed (Table 3). In addition, Collins and Cruickshank (2013) suggested that NJ and other tree inference methods are indeed poor proxies to infer specimen identifications. A similar occurrence can be found in the grouping pattern of *Lu. longipalpis* and *Lu. cruzi* in a study in Brazil (de Pinto et al., 2015). Furthermore, queries of the *Pa. abonnenci* sequences on NCBI and BOLD databases returned *Pa. shannoni* and *Pa. bigeniculata*, respectively, as the closest match with percentage identity <94%, which is low for concluding on definite species identification. Occurrence like this is likely to reduce as the reference library becomes more populated with additional sequences from sand flies species across the taxonomic spectrum of this group.

In contrast, high intraspecific divergence (Table 2) and deep split (Figure 2) observed in *Lu. cruciata* and *Br. mesai* could be a result of genetic isolation or misidentification. We particularly suspected cases of possible misidentifications in *Br. mesai* samples from Colombia retrieved from GenBank. These samples clustered distinctly from the *Br. mesai* samples collected in the current study (Figure 2) and showed a high mean intraspecific divergence of 9.12% and maximum pairwise intraspecific divergence of 15.56% (Table 2) compared to the 0.61% and 0.94% from the sequences generated in the current study (Appendix S1). Identification of *Br. mesai* in the present study is not in doubt as this is a common and abundant species in Quintana Roo (Rodríguez‐Rojas & Rebollar‐Téllez, 2017); likewise, the misidentification of KR907864 and GU909506 is quite plausible given that the sequences were unpublished (Table S1), and submitting authors may have not given careful consideration to the morphological identification. We suspected the specimens are likely from species typical of Colombia with no current representation on public databases. Identification queries on BOLD's and GenBank search engines returned species with low percentage similarity. *Lu. cruciata* sample (BOLD ID: HNLUZ014‐17) retrieved from BOLD also has high sequence divergence with sequences obtained in the current study (Table 2). However, all *Lu. cruciata* sequences clustered together in the NJ tree, albeit with a deep split (Figure 2). Morphological examination of BOLD ID: HNLUZ014‐17, based on the photograph uploaded in BOLD, is consistent with *Lu. cruciata* supporting the conclusion that the divergence observed could likely be due to genetic isolation as a result of differing geographical locations or the presence of cryptic species (Gutiérrez et al., 2014).

A species is considered as successfully delimited using ABGD when all its members belong to the same predicted group and no other sequences were added to it (Puillandre et al., 2012). The DNA barcode analyses performed using the automatic partitioning by ABGD allowed the correct discrimination of almost 100% of all previously morphologically identified species. With eight Molecular Operational Taxonomic Units (MOTUs) identified, all morphologically identified species grouped together, except for *Pa. shannoni* that split into two groups. The classification of *Da. beltrani* and *Da. steatopyga* in the present study is tentative because the species discrimination cannot be reliably accomplished using female samples alone as the taxonomic keys of these species are incomplete, and females of *Da. steatopyga* and *Da. beltrani* have not been adequately morphologically described.

## 5 CONCLUSION

In conclusion, our results are congruent with the argument that the DNA barcoding approach is a valuable tool for species identification sand flies. This study augmented available DNA barcoding data for phlebotomine sand fly species and provided three unique BINs that were not previously found in BOLD, contributing toward the establishment of a reliable reference DNA barcode library for phlebotomine sand fly identification in Mexico. Certain taxa might, however, require additional genetic markers in addition to *cox1* for correct delimitation. Limited representation of species from different geographical regions in Quintana Roo and Mexico in the present study also warrants an expanded study to provide a comprehensive national barcode reference library for phlebotomine sand flies species in the region.

## CONFLICT OF INTERESTS

The authors declare that they have no competing interest.

## AUTHORS' CONTRIBUTION

AAA and MAR‐P designed and conceived the study. NAF‐S, NT‐G, HH‐J, PCM‐A, WAP‐P, and JJR‐R collected and identified the samples. AAA and NAF‐S did the molecular analysis. AAA and LMH‐T interpreted the data. NT‐G, HH‐J, and MAR‐P coordinated the study. AAA wrote the initial manuscript draft. All authors read and approved the final manuscript.

## ETHICS APPROVAL AND CONSENT TO PARTICIPATE

The study involved the use of adult sand flies collected as part of regular entomological surveillance by local health authorities. No ethics committee approval is needed for such work.

## CONSENT FOR PUBLICATION

Not applicable.

## Data Availability

All sequences generated in the study and information about additional sequences downloaded from GenBank and BOLD databases are provided in Table S1. Data generated from the study have been deposited and available in GenBank with the accession numbers MK851242–MK851285 and on BOLD under the project AAASF.
