# Supplementary figures and images for: Identification of phlebotomine sand flies (Diptera: Psychodidae) from leishmaniasis endemic areas in southeastern Mexico using DNA barcoding
Source: Ecol Evol. 2019 Nov 21;9(23):13543–54. doi: 10.1002/ece3.5811 (PMC6912917; doi:10.1002/ece3.5811)

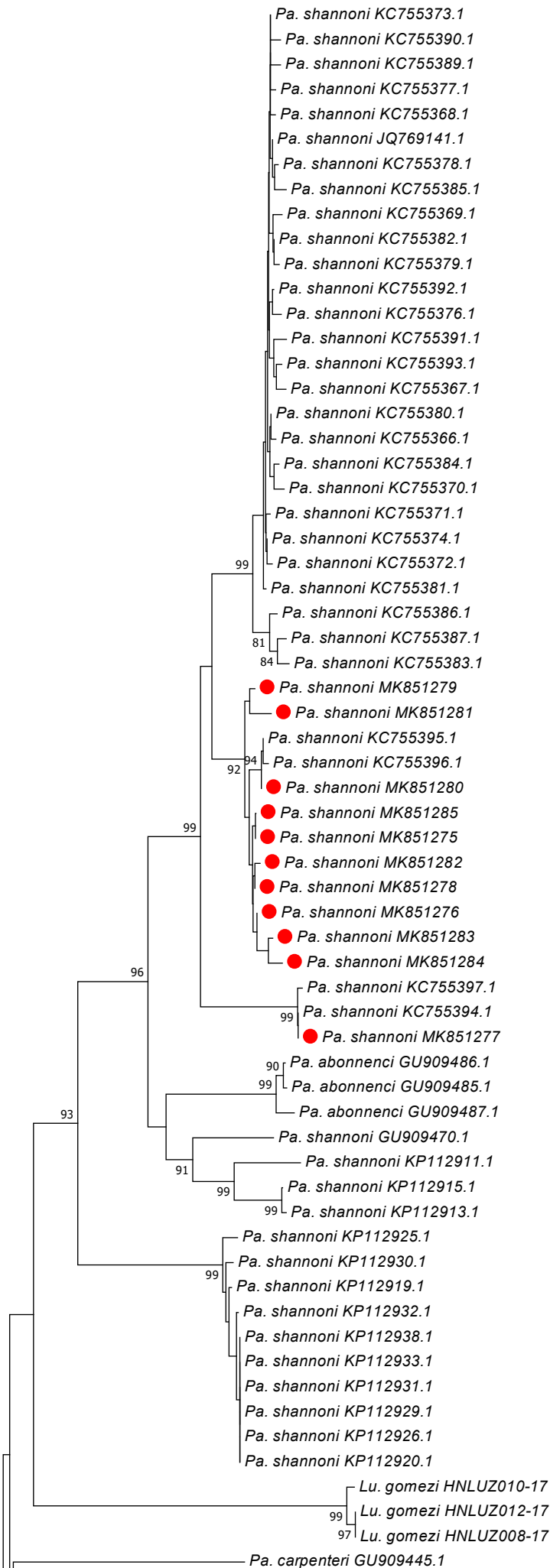

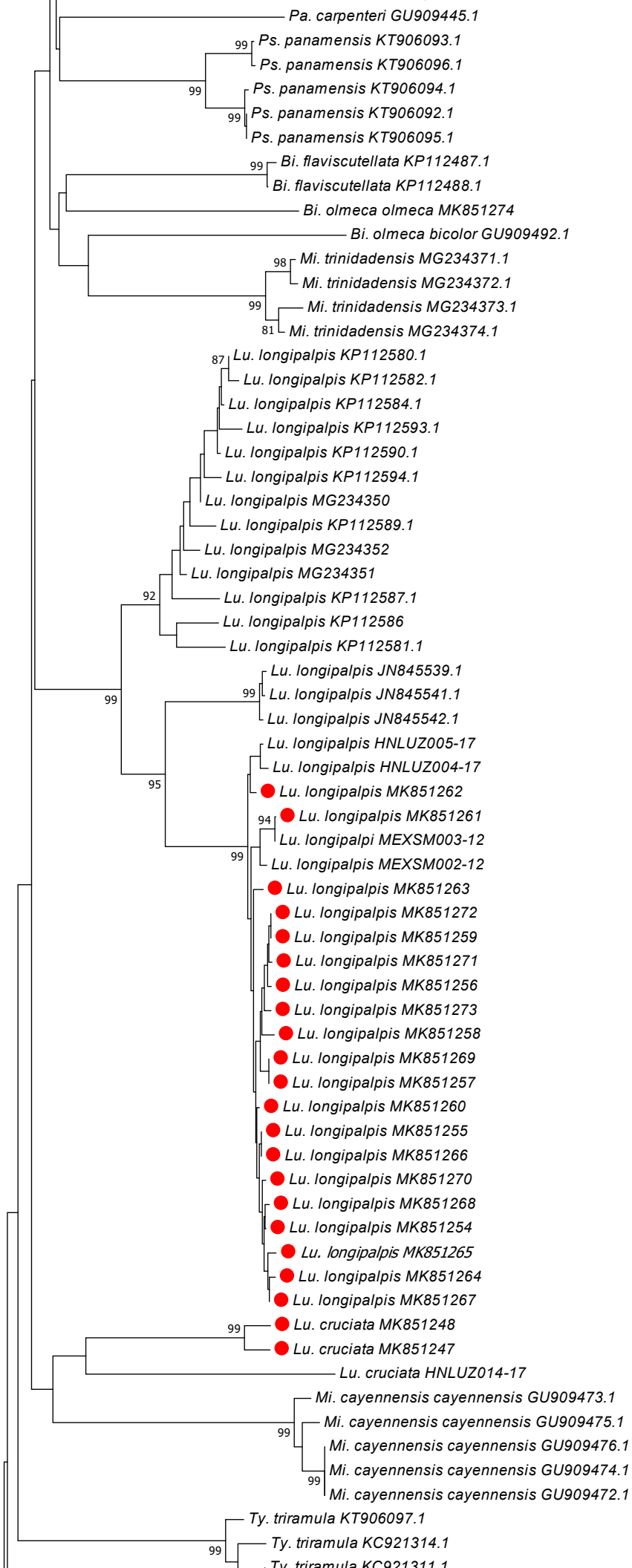

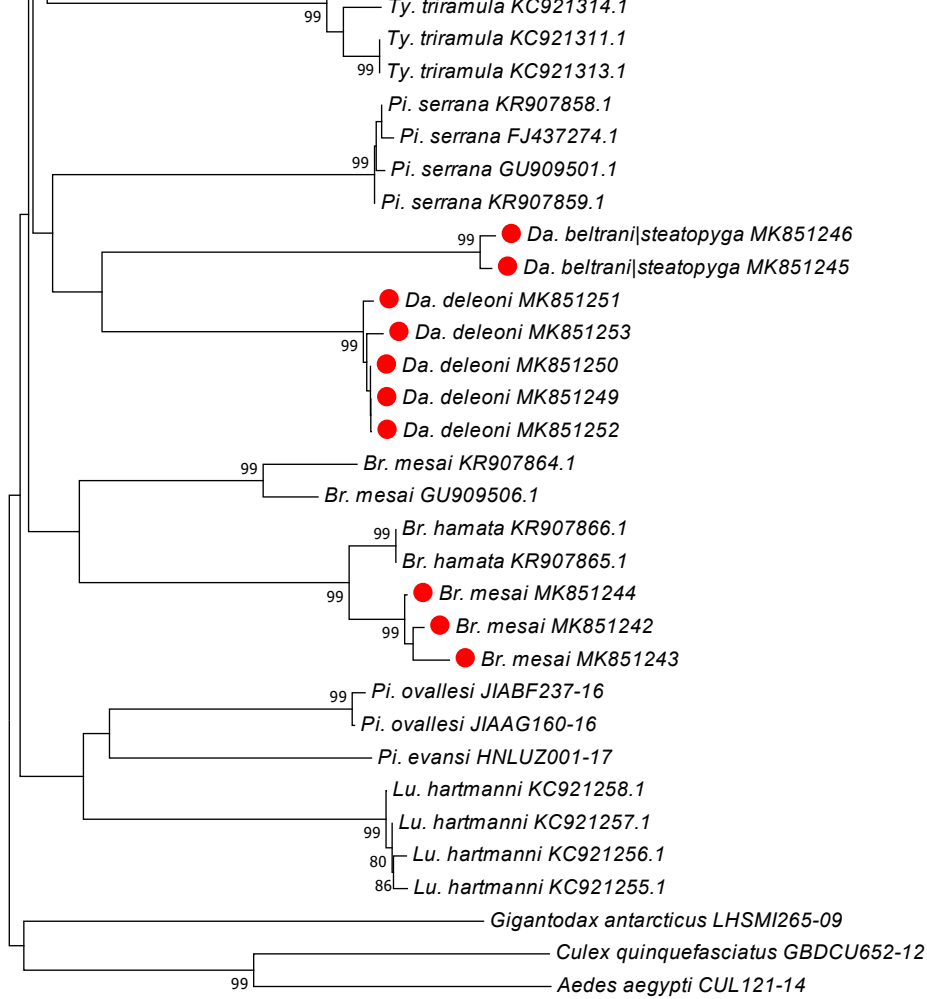

Supplement: Supplementary file 1 [file ECE3-9-13543-s001.pdf]

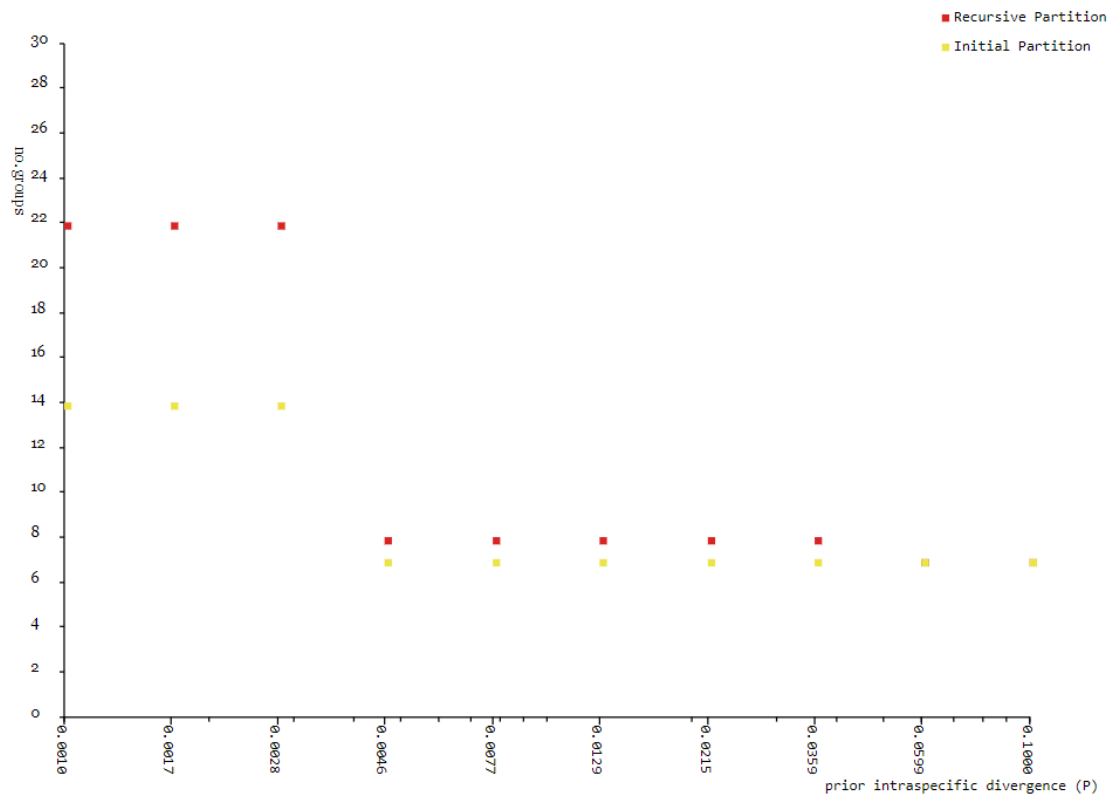

Supplement: Supplementary file 6 [file ECE3-9-13543-s006.pdf]
