# Supplementary material for: Identification of phlebotomine sand flies (Diptera: Psychodidae) from leishmaniasis endemic areas in southeastern Mexico using DNA barcoding
Source: Ecol Evol. 2019 Nov 21;9(23):13543–54. doi: 10.1002/ece3.5811 (PMC6912917; doi:10.1002/ece3.5811)
Supplement: Supplementary file 5 [file ECE3-9-13543-s005.docx]

**Appendixes**

**Appendix S1**: Mean and maximum sequence divergence (K2P model) of phlebotomine sand fly collected in Mexico.

| Species | Number of DNA barcodes (n) | Mean sequence Divergence (%) | Maximum pairwise divergence (%) | Number of haplotypes  (h) |
| --- | --- | --- | --- | --- |
| 1. *Bichromomyia olmeca olmeca* | 1 | N/A | N/A | 1 |
| 1. *Brumptomyia mesai* | 3 | 0.61 | 0.94 | 2 |
| 1. *Dampfomyia beltrani/Da. steatopyga* | 2 | 0.3 | 0.61 | 2 |
| 1. *Dampfomyia deleoni* | 5 | 0.12 | 0.62 | 2 |
| 1. *Lutzomyia cruciata* | 2 | 1.22 | 1.6 | 2 |
| 1. *Lutzomyia longipalpis* | 20 | 0.39 | 1.08 | 8 |
| 1. *Psathyromyia shannoni* | 11 | 1.13 | 4.5 | 6 |

**Appendix S2**: Kimura-2-Parameter pairwise interspecific sequence divergence (%)

|  | *Br.*  *mesai* | *Da. beltrani/ steatopyga* | *Lu. cruciata* | *Da. deleoni* | *Lu. longipalpis* | *Pa. shannoni* | *Bi olmeca olmeca* |
| --- | --- | --- | --- | --- | --- | --- | --- |
| *Brumptomyia mesai* |  |  |  |  |  |  |  |
| *Dampfomyia beltrani/ Da. steatopyga* | 18.84 |  |  |  |  |  |  |
| *Lutzomyia cruciata* | 14.11 | 16.09 |  |  |  |  |  |
| *Dampfomyia deleoni* | 14.52 | 11.59 | 16.33 |  |  |  |  |
| *Lutzomyia longipalpis* | 16.07 | 17.77 | 12.16 | 15.78 |  |  |  |
| *Psathyromyia shannoni* | 17.75 | 19.29 | 14.43 | 18.70 | 12.93 |  |  |
| *Bichromomyia olmeca olmeca* | 16.85 | 18.50 | 13.78 | 15.36 | 13.48 | 14.86 |  |

**Figure legend:**

**Appendix S3**: Number of groups among the 44 *cox1* sequences of phlebotomine sand flies collected in Quintana Roo, Mexico based on the values of prior intraspecific divergence found by the ABGD (Automatic Barcode Gap Discovery) analysis as potential barcode gaps using a range of 0.001 to 0.1 for prior intraspecific divergence

**Additional files**

Additional file 1 (Table S1): Name and GenBank/BOLD ID of sequences used for the phylogenetic analysis.

Additional file 2 (Table S2): Nucleotide composition of the 44 phlebotomine sand fly cox1 sequences retrieved from Quintana Roo.

Additional file 3 (Table S3): Pairwise distance analysis (K2P model) of collected sequences in the current study.

Additional file 4 (Figure S1): Expanded Neighbour-Joining tree of *cox1* sequence divergences (K2P) of the 157 individuals analysed. Samples collected in the present study are highlighted in red. Only bootstrap values higher than 80 are shown in the tree.
